# Prospective development and validation of an objective classification for difficult facemask ventilation: the MASCAN score

**DOI:** 10.1111/anae.70262

**Published:** 2026-06-05

**Authors:** Viktor A. Wünsch, Hannes Bommes, Milan Piltan, Sofia Germer, Christopher Gundler, Thorsten Dohrmann, Vera Köhl, André Dankert, Linda Krause, Christian Zöllner, Philipp Breitfeld, Martin Petzoldt

**Affiliations:** ^1^ Department of Anaesthesiology, Centre for Anaesthesiology and Intensive Care Medicine University Medical Centre Hamburg‐Eppendorf Hamburg Germany; ^2^ Institute of Applied Medical Informatics University Medical Centre Hamburg‐Eppendorf Hamburg Germany; ^3^ Institute of Medical Biometry and Epidemiology University Medical Centre Hamburg‐Eppendorf Hamburg Germany

**Keywords:** airway management, facemask ventilation, respiratory system

## Abstract

**Introduction:**

Difficult facemask ventilation is an entity that lacks a robust definition, leading to inconsistent identification in clinical practice and research. The aim of this study was to develop and validate an objective classification and numeric score for difficult facemask ventilation.

**Methods:**

Four hundred patients who required tracheal intubation for ear, nose and throat or maxillofacial surgery participated in this prospective single centre study. After induction of anaesthesia, facemask ventilation was attempted using pressure‐controlled mechanical ventilation. The primary outcome was difficult facemask ventilation documented as an alert in the patient health records. An independent observer assessed for potential indicators of difficult facemask ventilation: two‐handed grip; oral airway use; jaw thrust; senior consultant anaesthetist taking over; conversion to manual rescue ventilation; peripheral oxygen saturation drop; tidal volume; and leak fraction.

**Results:**

Difficult facemask ventilation occurred in 43 (10.8%) patients. Five eligible indicators were selected by cross‐validated least absolute shrinkage selector operator regression and used to develop a multivariable logistic regression model (MASCAN model) and score (MASCAN score). These indicators were: two‐handed grip; oral airway use; jaw thrust; tidal volume ≤ 2 ml.kg^‐1^; and peripheral oxygen saturation drop ≥ 10%. The area under the receiver operating characteristic curve was 0.96 (95%CI 0.93–0.98) for the MASCAN model and 0.94 (95%CI 0.91–0.98) for the MASCAN score.

**Discussion:**

The MASCAN score is an objective, data‐driven classification for difficult facemask ventilation with clearly defined thresholds that may improve airway documentation and inform future airway management.

## Plain Language Summary

**What we did:**

Researchers studied 400 patients having head and neck surgery. After the patients were given an anaesthetic, doctors used a facemask to ventilate them. The researchers looked for signs that facemask ventilation was difficult, such as using two hands to hold the mask, using an oral airway tool, doing a jaw thrust, low airflow into the lungs or seeing oxygen levels drop. They then created a new classification system called the MASCAN score to help measure how difficult facemask ventilation was.

**Why did we do it:**

Doctors sometimes find it hard to ventilate patients with a facemask during anaesthesia. There has not been a clear and reliable way to measure and document this problem. The researchers wanted to create a simple and objective system that doctors could use to communicate difficult facemask ventilation more easily and accurately.

**What we found:**

About 1 in 10 patients had difficult facemask ventilation. The researchers found five important signs that reliably describe difficult facemask ventilation. These include using two hands to hold the mask, using an oral airway tool, doing a jaw thrust, low airflow into the lungs and a drop in oxygen levels. The new MASCAN score was very good at classifying difficult facemask ventilation and may help doctors improve their record keeping and future patient care.

## Introduction

Facemask ventilation is a key component of airway management in anaesthesia, intensive care and emergency medicine. Difficult facemask ventilation can result in life‐threatening hypoxaemia, especially if accompanied by other airway management challenges [1, 2, 3, 4].

Despite its ubiquity and clinical importance, difficult facemask ventilation remains poorly classified, leading to inconsistent identification in clinical practice and research. Various definitions for difficult facemask ventilation have been reported that rely largely on subjective interpretation [1, 3, 5, 6, 7, 8, 9, 10, 11, 12, 13, 14]. These classification systems do not provide numeric scores or evidence‐based thresholds and might lead to inconsistency and variance. Accordingly, reported incidences of difficult facemask ventilation vary widely between 0.08% and 15% depending on the definition used [15, 16].

As a history of difficult airway management is the strongest predictor of future difficulty [17, 18, 19], a reproducible, quantitative classification system might improve the identification and documentation of difficult facemask ventilation. This study aimed to develop and validate such a quantitative classification system through evidence‐based selection of parameters and empirical thresholds.

## Methods

The single‐centre prospective observational MASCAN study [20] was approved by the Ethics Committee of the Medical Association of Hamburg and conducted in accordance with the revised Declaration of Helsinki [21]. The study took place between November 2022 and May 2023. This study represents an additional analysis that was conducted and reported in adherence with the TRIPOD statement [22]. The need for supplementary approval was waived by the ethics committee.

Patients were assessed for study eligibility if they were aged ≥ 18 y and scheduled for general anaesthesia with facemask ventilation and subsequent tracheal intubation for elective ear, nose and throat or maxillofacial surgery. Patients underwent a structured airway evaluation that included: medical conditions; review of previous airway management; upper lip bite test; Wilson score; and Simplified Airway Risk Index [16, 23, 24, 25]. Transnasal videoendoscopy was performed, if appropriate [26, 27]. Further details are provided elsewhere [20].

The optimal facemask size was identified by placing different sized silicone masks (Armstrong Medical, Coleraine, UK) on the patient's face and selecting the mask that best covered the nose and mouth without overlapping the eyes, as determined by the airway manager. After the loss of eyelid reflex, facemask ventilation was attempted using pressure‐controlled ventilation delivered by a mechanical ventilator (Perseus^®^ A500, Draeger Medical, Lübeck, Germany) with the following settings: inspiratory pressure 15 cmH_2_O; respiratory rate 10 breaths.min^‐1^; PEEP 0 cmH_2_O; and I:E ratio 1:2 [28, 29, 30]. A fraction of inspired oxygen of 1.0 at 15 l.min^‐1^ was delivered. Facemask ventilation was optimised by adjusting the head and neck position at the discretion of the airway operator. Rocuronium bromide 0.6–1.2 mg.kg^‐1^ (predicted body weight) was administered to all patients after evaluation of facemask ventilation. If the quality of facemask ventilation was not regarded as acceptable by the airway operator, a total dose of rocuronium 1.2 mg kg^‐1^ predicted body weight was ultimately administered, if appropriate. A train‐of‐four count of 0 (ToFscan^®^, Draeger Medical, Lübeck, Germany) was checked before tracheal intubation was performed using a direct laryngoscope (Dahlhausen LED Stainless Steel, Cologne, Germany). Airway operators were resident or consultant anaesthetists; their age and duration of professional experience were recorded.

Eight potential indicators of difficult facemask ventilation, either manoeuvres to counteract difficulties or clinical signs of deterioration, were preselected by literature search, previous studies and clinical experience [1, 3, 5, 6, 7, 8, 9, 10, 11, 12, 13, 15]: two‐handed grip; oral airway use; jaw thrust; a senior consultant anaesthetist taking over; conversion to manual facemask ventilation (with inspiratory pressure > 15 cmH_2_O permissible); peripheral oxygen saturation (SpO_2_) drop (%) during facemask ventilation (between the end of pre‐oxygenation and facemask removal for the purpose of tracheal intubation); tidal volume (ml.kg^‐1^ predicted body weight, maximum value observed); and leak fraction (leakage volume per tidal volume, lowest value observed). Potential indicators and study outcomes were assessed systematically by an independent observer not involved in patient care, with indicators recorded before the administration of a neuromuscular blocking drug. Tidal and leakage volumes were recorded from the ventilator while the SpO_2_ values were recorded from the monitoring system (Infinity^®^ Delta, Dräger, Lübeck, Germany).

The primary outcome measure was difficult facemask ventilation, defined as an alert documented in the patient health record by the airway operator after the difficulty was encountered. This alert was issued based on the clinical consideration of the airway operator within routine practice and not on predefined criteria. Secondary outcomes were impossible facemask ventilation and airway‐related adverse events. Impossible facemask ventilation was defined as absence of end‐tidal carbon dioxide measurement and lack of perceptible chest wall movement despite use of airway adjuncts, administration of neuromuscular blocking drugs and assistance of additional personnel [8, 9]. An airway‐related adverse event was regarded as any of airway trauma including bleeding and dental injury; glottic swelling; hypotension (systolic blood pressure < 70 mmHg); hypoxaemia (SpO_2_ ≤ 93%); laryngospasm and pulmonary aspiration.

We expected an incidence of 11% for difficult facemask ventilation based on pooled data from a systematic Cochrane review [16] and our own pre‐trial experience [17, 25, 26, 31]. As this is the first study of its kind, data for a priori sample size estimation were unavailable. All 400 patients from the MASCAN study were included. We performed regularised regression with stability selection to balance and control the number of co‐variables for the final multivariable regression model and avoid overfitting [32, 33, 34].

We used least absolute shrinkage selector operator (LASSO) regression with a generalised linear model (R package ‘glmnet’) to select eligible co‐variables out of the eight candidate indicators for difficult facemask ventilation [35, 36]. We used resampling for variable selection to control for false discoveries [33]. The shrinkage parameter λ was determined by ten‐fold cross‐validation and optimised for binomial deviance, resulting in one distinct optimal model. To reduce variability, we performed 100 repetitions of a ten‐fold cross‐validated LASSO regression and calculated the selection rate (percentage of cross‐validations in which the variable was selected, i.e. the β‐coefficient was not shrunk to zero) for each indicator as reported previously [37]. We calculated the median cross‐validated area under the receiver operating characteristic curve (AUROC) with IQR as reported previously [37]. Only indicators with a selection rate ≥ 60% were used as co‐variables for the fitting of the final multivariable logistic regression model (the MASCAN model) for the classification of difficult facemask ventilation based on the selected co‐variables.

In addition, we built an easy‐to‐use MASCAN score. During score development, we categorised continuous variables using a data‐driven approach based on pre‐specified zero points in conjunction with β‐coefficients, which we obtained by multivariable regression analysis. We divided β‐coefficients by two and rounded them to integer numbers (for binary variables, giving a point value of +1 per co‐variable) or one decimal place (for continuous variables, giving a conversion factor) [37]. Zero points for continuous variables rely on recognised, clinically meaningful baseline conditions that were identified by literature search and clinical considerations (initial SpO_2_ after pre‐oxygenation to determine any subsequent ‘SpO_2_ drop’ and 12 ml.kg^‐1^ predicted body weight for ‘tidal volume’) [28, 29, 30]. Finally, we categorised continuous variables and assigned integer point values by adding multitudes of the conversion factor to the designated zero points as reported previously [26, 38, 39].

We used sensitivity, specificity, positive and negative predictive value, and positive and negative likelihood ratios to determine clinically relevant thresholds of the final MASCAN score [40]. We calculated the Youden index of the MASCAN score to determine optimal thresholds based on the maximal Youden index, while taking clinical utility into account. We calculated the AUROCs with 95%CIs for the MASCAN model and score. Diagnostic performance was regarded as poor with an AUROC of 0.51–0.69, fair at 0.7–0.79, good at 0.8–0.89 and excellent at 0.9–0.99 [41]. To evaluate model calibration, we plotted a GiViTI calibration belt (R package ‘givitiR’, version 1.3) [42, 43, 44].

Post‐hoc sensitivity analysis was performed to test the robustness and reliability of the final MASCAN model, assuming that adding user professional experience would not alter model performance and goodness of fit, as determined by the AUROC and Akaike information criterion [45]. Furthermore, we calculated a linear model with natural cubic splines to evaluate the association between the leak fraction and tidal volume.

We checked metric variables for normal distribution by visual inspection of histograms. Statistical analyses were performed using R version 4.3.1 (R Foundation for Statistical Computing, Vienna, Austria) and SPSS statistics version 27 (IBM Inc., Armonk, NY, USA).

## Results

**Figure 1:**
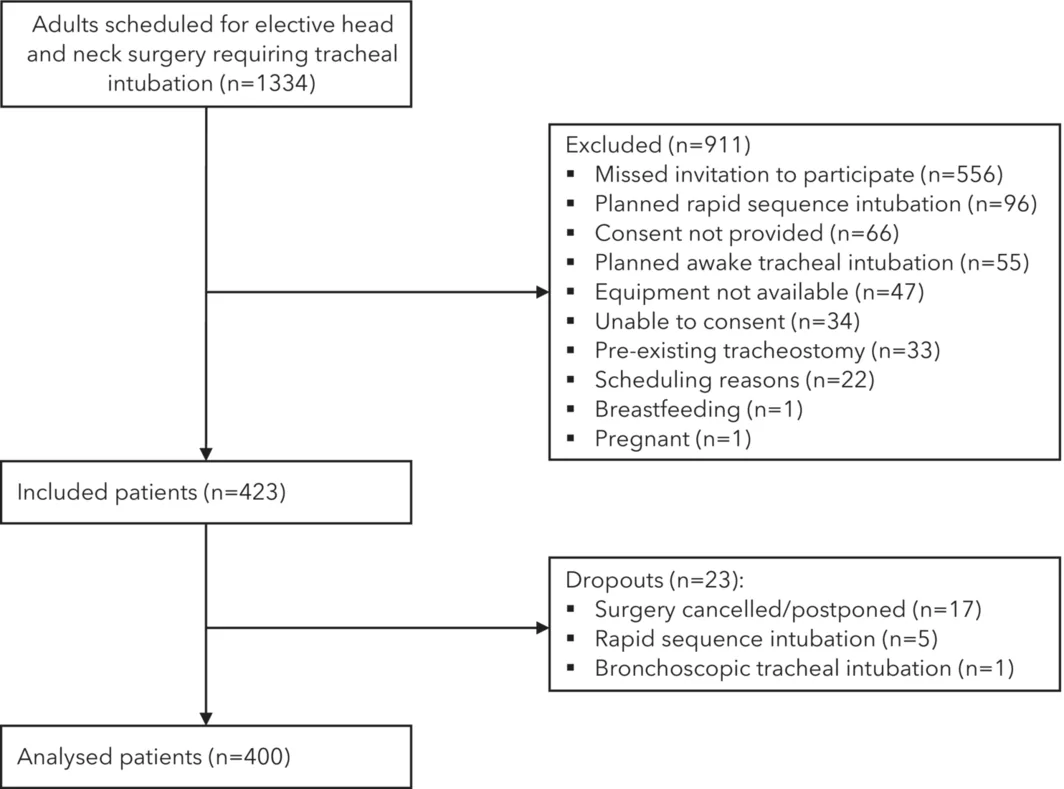
Study flowchart.

In the MASCAN study [20], 1334 patients were assessed for eligibility and 423 were enrolled (Fig. 1), with 400 contributing data for analysis (Table 1). Airway operators had a median (IQR [range]) age of 30 (27–34 [25–55]) y and professional experience of 30 (4–60 [1–286]) months. Difficult facemask ventilation occurred in 43 (10.8%) patients and impossible facemask ventilation occurred in three (0.8%). Airway‐related adverse events were observed in 10 (2.5%) patients (Table 2). There were no missing values for the variables required for model fitting.

**Table 1:** Characteristics of the study cohort. Values are median (IQR [range]) or number (proportion).

	All patients	Easy facemask ventilation*	Difficult facemask ventilation*
	n = 400	n = 357	n = 43
Age; y	47 (31–63 [18–88])	47 (31–62 [18–88])	53 (35–70 [26–80])
BMI; kg.m^‐2^	25 (23–28 [16–51])	25 (22–28 [16–51])	29 (25–34 [20–51])
Sex; female	183 (45.8%)	169 (47.3%)	14 (32.6%)
ASA physical status			
1	93 (23.3%)	87 (24.4%)	6 (14.0%)
2	225 (56.2%)	202 (56.6%)	23 (53.5%)
3	81 (20.3%)	68 (19.0%)	13 (30.2%)
4	1 (0.2%)	‐	1 (2.3%)
**Type of surgery**			
Dentoalveolar	29 (7.3%)	25 (7.0%)	4 (9.3%)
Ear, nose, sinus	163 (40.8%)	145 (40.6%)	18 (41.8%)
Endo‐/exocrine glands	24 (6.0%)	22 (6.2%)	2 (4.7%)
Mandibular	39 (9.8%)	35 (9.8%)	4 (9.3%)
Microlaryngoscopy	23 (5.8%)	21 (5.9%)	3 (7.0%)
Neck and maxillofacial	58 (14.5%)	51 (14.3%)	7 (16.2%)
Pharyngolaryngeal	53 (13.3%)	50 (14.0%)	3 (7.0%)
Other	10 (2.5%)	8 (2.2%)	2 (4.7%)

* Refers to whether the primary study outcome of ‘difficult facemask ventilation’ was achieved or not.

**Table 2:** Outcome measures and predictor variables of 400 patients with or without difficult facemask ventilation. Values are number (proportion) or median (IQR [range]).

	All patients	Easy facemask ventilation*	Difficult facemask ventilation*
	n = 400	n = 357	n = 43
**Outcome measures**			
Difficult facemask ventilation	43 (10.8%)	‐	43 (100%)
Impossible facemask ventilation	3 (0.8%)	‐	3 (7.0%)
Airway‐related adverse events	10 (2.5%)	9 (2.5%)	1 (2.3%)
**Predictor variables**			
Two‐handed grip	118 (29.5%)	77 (21.6%)	41 (95.3%)
Oral airway	102 (25.5%)	62 (17.4%)	40 (93.0%)
Jaw thrust	82 (20.5%)	44 (12.3%)	38 (88.4%)
Tidal volume; ml.kg^‐1^ ^†^	9.0 (5.7–11.9 [0–21.9])	9.3 (6.5–12.4 [0.2–21.9])	3.3 (0.7–5.9 [0–17.8])
SpO_2_ drop; %	0 (0–0 [0–17])	0 (0–0 [0–7])	0 (0–1 [0–17])
Senior consultant taking over	6 (1.5%)	2 (0.6%)	4 (9.3%)
Manual rescue ventilation	9 (2.3%)	3 (0.8%)	6 (14.0%)
Leak fraction	0.15 (0.02–0.41 [0–1.00])	0.13 (0.02–0.37 [0–0.99])	0.53 (0.11–0.86 [0–1.00])

* Refers to whether the primary study outcome ‘difficult facemask ventilation’ was achieved or not.

^†^ Predicted body weight.

**Figure 2:**
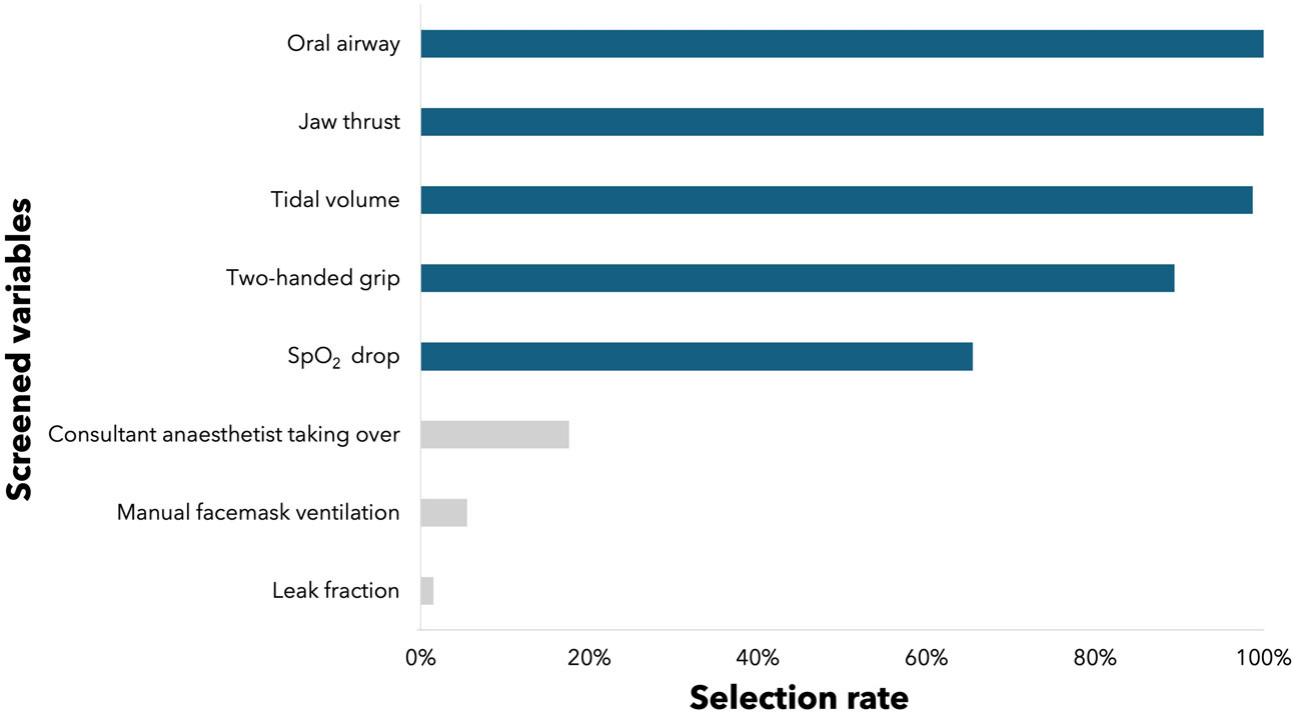
Selection rate of indicators for difficult facemask ventilation during 100‐times repeated ten‐fold cross‐validated LASSO regressions. Only important variables (cut‐off values of ≥ 60%) were selected for the fitting of the final multivariable model (blue bars). Numerical data are provided in online Supporting Information Table S3.

One hundred times repeated, ten‐fold cross‐validated LASSO regression selected five eligible co‐variables for model fitting out of the eight potential indicators for difficult facemask ventilation (Fig. 2). The diagnostic performance of these 1000 LASSO regression models was excellent with a median (IQR) cross‐validated AUROC of 0.96 (0.93–0.98) (online Supporting Information Figure S1). These five indicators (two‐handed grip, oral airway use, jaw thrust, tidal volume (ml.kg^‐1^ predicted body weight) and SpO_2_ drop (%)) were selected in > 60% of these LASSO regressions (Fig. 2). These indicators were used for fitting of the final multivariable prediction model (Table 3). Three other variables (senior consultant anaesthetist taking over, conversion to manual rescue ventilation and leak fraction) achieved a selection rate < 60% and were therefore not used for model fitting.

**Table 3:** MASCAN model and score for the prediction of difficult facemask ventilation.

Co‐variables	MASCAN model	Zero points and conversion factor	MASCAN score point values*
	β‐coefficients (95%CI)		
Intercept	‐3.93 (‐6.00 to ‐2.39)	‐	‐2
Two‐handed grip	1.55 (‐0.19–3.62)	‐	+1
Oral airway	2.23 (0.94–3.81)	‐	+1
Jaw thrust	1.26 (0.03–2.64)	‐	+1
Tidal volume; ml.kg^‐1^ ^†^	‐0.21 (‐0.35 to ‐0.09)	ZP: 12 ml.kg^‐1^ Conversion factor: ‐0.1 per ml.kg^‐1^	+1 (if ≤ 2 ml.kg^‐1^)
SpO_2_ drop; %	0.19 (‐0.04–0.54)	ZP: baseline value pre‐induction Conversion factor: 0.1 per 1% drop	+1 (if drop ≥10%)
Range (without intercept)			0–5
AUROC (95%CI)	0.96 (0.93–0.98)		0.94 (0.91–0.98)

ZP, designated zero points for continuous variables [28, 29, 30]; CF, conversion factor; AUROC, area under the receiver operating characteristic curve. ZP plus a multitude of the conversion factor gives the final point value for continuous variables [26, 38, 39].

* β‐coefficients were divided by two and rounded to integer numbers (binary variables) or one decimal place (continuous variables, giving the conversion factor);

^†^ Predicted body weight.

**Figure 3:**
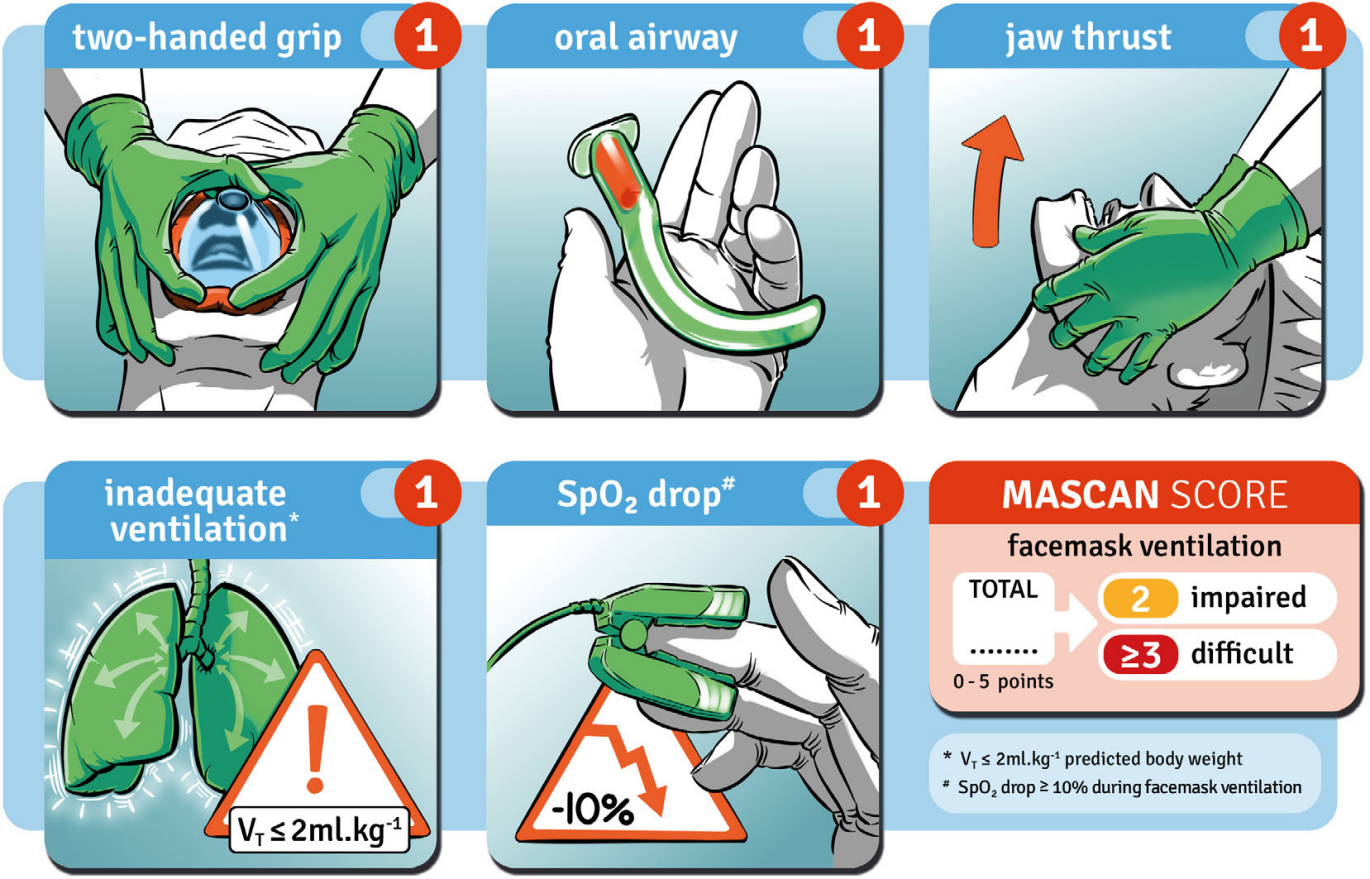
The MASCAN score for the classification of difficult facemask ventilation. Illustration by Rasmus Borkamp, Hamburg, Germany.

To build the MASCAN score, continuous variables were categorised based on predefined rules outlined in the methods section. Our analysis revealed that thresholds of ≤ 2 ml.kg^‐1^ for tidal volume and ≥ 10% for drop in SpO_2_ were optimal based on the derived ß‐coefficients and designated zero points (Fig. 3). The final MASCAN score assigned a point value of +1 to each indicator for a total of 0–5 points (Table 3). Total point values of 2 and 3 achieved the highest Youden indices: 0.81 (95%CI 0.73–0.87) and 0.81 (95%CI 0.71–0.89), respectively. A score of 2 points had a sensitivity of 0.95 (95%CI 0.99–1.00) and specificity of 0.86 (95%CI 0.82–0.89) and was considered optimal for the label of ‘impaired facemask ventilation’ (yellow flag) due to its high sensitivity. A score of 3 points had a sensitivity 0.91 (95%CI 0.81–0.98) and specificity of 0.90 (95%CI 0.87–0.93) and was considered an optimal threshold for the label of ‘difficult facemask ventilation (red flag)’ due to its high specificity (online Supporting Information Table S1).

The MASCAN model and score showed excellent diagnostic performance with an AUROC of 0.96 (95%CI 0.93–0.98) for the MASCAN model and 0.94 (95%CI 0.91–0.98) for the MASCAN score, respectively (online Supporting Information Figure S2). The GiViTI calibration belt fitted well in the entire range of probabilities, indicating good model calibration (online Supporting Information Figure S3).

Post hoc sensitivity analysis revealed that model performance and goodness of model fit, expressed as AUROC and Akaike information criterion of the MASCAN model, did not improve when user professional experience level was added to the MASCAN model (online Supporting Information Table S2). The linear model with natural cubic splines showed a clear negative correlation between the leak fraction and tidal volume (online Supporting Information Figure S4). However, even high mean leakage volumes ≥ 60% were still associated with sufficient mean tidal volumes > 2 ml.kg^‐1^ predicted body weight.

## Discussion

The MASCAN score originates from a large, prospective development and validation study and showed excellent diagnostic performance and calibration. The MASCAN score comprises five simple objective clinical indicators that are assessed routinely in daily clinical practice. A score of two supports documenting an ‘impaired facemask ventilation’ alert (yellow flag) and a score of three or more supports documenting a ‘difficult facemask ventilation’ alert (red flag) in patients' health records. These documented scores may improve communication between healthcare professionals, assist with anticipating and preventing adverse events and inform the need for awake tracheal intubation.

The incidence of difficult facemask ventilation was 10.8%, which is consistent with the median incidence from pooled data from a systematic Cochrane review (11%) [16]. Definitions of difficult facemask ventilation have included adjectives such as inadequate [3, 8, 10]; unstable [3, 8, 10]; poor [5]; or insufficient [5]. These descriptors are vague and influenced strongly by subjective interpretation [8]. Surrogates for impaired facemask seal [1, 7, 11, 13] or upper airway obstruction have also been used in previous studies, such as excessive resistance [1]; altered capnography waveforms [14]; oral airway use [5, 8, 10, 12, 13]; nasal airway use [12]; jaw thrust [3]; and other additional manoeuvres [5].

The clinical indicators within the MASCAN score are objective, as they reflect distinct binary actions (application of oral airway, jaw thrust and two‐handed grip) or directly measured physiological parameters (tidal volume and SpO_2_). There is little reliance on the subjective interpretation of the user. Three of the indicators are manoeuvres to counteract difficulty while the other two reflect deterioration of ventilation or oxygenation. While the requirement of two people to perform facemask ventilation has been used as an indicator of difficulty in previous studies [8, 10, 11, 12], the seniority and nature of the assistance are often unclear. To account for this, we used ‘two‐handed grip’ and ‘senior consultant anaesthetist taking over’ as two different candidate predictors for model development.

Gas leakage originates from insufficient facemask seal and has also been proposed as an indicator of difficult facemask ventilation [1, 3, 11]. Our data show that gas leakage itself is not predictive of difficult facemask ventilation if tidal volumes > 2 ml.kg^‐1^ predicted body weight are measured. Adequate tidal volumes may still be achieved despite gas leakage and are aided by increased gas flow rates. In this study, pressure‐controlled ventilation with a peak pressure of 15 cmH_2_O was chosen because it provides the highest probability of acceptable facemask ventilation while limiting the risk of gastric insufflation [28, 30, 46]. A respiratory rate of 10 breaths.min^‐1^ was chosen to avoid excessive minute ventilation [28, 29, 30].

This study has some limitations. As standards, equipment, skills and surgical populations differ between institutions, the findings of this single‐centre study should be generalised with caution. While ethnicity was not recorded, the majority of patients were likely White European and all patients were attending for elective ear, nose and throat or maxillofacial surgery. The study did not investigate if clinical implementation of the MASCAN score improves patient outcomes. Neuromuscular blockade influences facemask ventilation. In this study, neuromuscular blocking drugs were administered after evaluating facemask ventilation. As it is common to use manual facemask ventilation in many institutions, external validation with manual ventilation would be valuable, as would evaluation with different doses and timings of neuromuscular blocking drugs.

In summary, the MASCAN score is a prospectively developed classification system for difficult facemask ventilation with excellent diagnostic performance and calibration. It is determined by objective parameters and enables observer‐independent, interoperable, reliable documentation in the patient's health records.

## Supporting information


**Figure S1.** Distribution of model AUROCs during LASSO regression obtained from repetitive cross‐validation.

**Figure S2.** Receiver operating characteristic curves for the classification of difficult facemask ventilation for the MASCAN model and MASCAN score.

**Figure S3.** GiViTI calibration belt of the MASCAN model outlining the agreement between the observed and expected probabilities of the primary outcome.

**Figure S4.** Leak fraction as a function of the tidal volume per linear model with natural cubic splines.

**Table S1.** Specification of the MASCAN score and decision thresholds with 95%CI obtained by 10,000‐fold bootstrapping.
**Table S2.** Goodness of fit and model performance when adding professional experience of the anaesthetist to the MASCAN model.
**Table S3.** Numerical data for Figure 2.
