# Supplementary material for: Prospective development and validation of an objective classification for difficult facemask ventilation: the MASCAN score
Source: Anaesthesia. 2026 Jun 5;81(10):1349–57. doi: 10.1111/anae.70262 (PMC13551291; doi:10.1111/anae.70262)
Supplement: Supplementary file 1 [file ANAE-81-1349-s005.docx]

**Table S1** Specification of the MASCAN score and decision thresholds with 95%CI obtained by 10,000-fold bootstrapping

| **Score** | | **Sensitivity** | **Specificity** | **PPV** | **NPV** | **LR+** | **LR-** |
| --- | --- | --- | --- | --- | --- | --- | --- |
| 0 | 1.00  (1.00–1.00) | | 0.00  (0.00–0.00) | 0.11  (0.11–0.11) | - | 1 (1.00–1.00) | - |
| 1 | 0.98  (0.93–1.00) | | 0.71  (0.66–0.76) | 0.29  (0.25–0.33) | 1.00  (0.99–1.00) | 3.35 (2.74–4.10) | 0.03 (0.00–0.11) |
| 2 | 0.95  (0.88–1.00) | | 0.86  (0.82–0.89) | 0.45  (0.39–0.52) | 0.99  (0.98–1.00) | 6.67 (4.93–9.15) | 0.05  (0.00–0.14) |
| 3 | 0.91  (0.81–0.98) | | 0.90  (0.87–0.93) | 0.53  (0.40–0.62) | 0.99  (0.98–1.00) | 9.25 (6.18–14.53) | 0.10  (0.02–0.21) |
| 4 | 0.35  (0.21–0.49) | | 0.99  (0.98–1.00) | 0.84  (0.65–1.00) | 0.93  (0.91–0.94) | 41.51 (10.67–inf.) | 0.66  (0.51–0.81) |
| 5 | 0.02  (0.00–0.07) | | 1.00  (1.00–1.00) | 1.00  (1.00–1.00) | 0.89  (0.89–0.90) | - | 0.98  (0.93–1.00) |

PPV, positive predictive value; NPV, negative predictive value; LR+, positive likelihood ratio; LR-, negative likelihood ratio; inf., infinite

**Table S2** Sensitivity analysis. Goodness of fit and model performance when adding anaesthetists professional experience to the MASCAN model

| **Co-variables** | **Modified MASCAN model β-coefficients (95% CI)** |
| --- | --- |
| (Intercept) | -4.24 (-6.41 – -2.58) |
| Oral airway | 2.14 (0.83 – 3.73) |
| Jaw thrust | 1.39 (0.12 – 2.82) |
| Two-handed grip | 1.56 (-0.20 – 3.64) |
| Tidal volume; ml kg^-1^ predicted body weight | -0.20 (-0.34 – -0.08) |
| S_P_O_2_ drop; % | 0.17 (-0.05 – 0.54) |
| Anaesthetists experience; month | 0.00 (0.00 – 0.01) |
| AUROC (95% CI) | 0.96 (0.93 – 0.98) |
| AIC with anaesthetists experience (sensitivity analysis) | 137.5 |
| AIC without anaesthetists experience (original MASCAN model) | 136.9 |

AUROC, area under the receiver operating characteristic curve; AIC, Akaike information criterion (lower values better)

**Table S3** Numerical data for Figure 2

| **Screened variables** | **Selection rate** |
| --- | --- |
| Oral airway | 100% |
| Jaw thrust | 100% |
| Tidal volume | 98.7% |
| Two-handed grip | 89.4% |
| SpO_2_ drop | 65.5% |
| Consultant anaesthetist taking over | 17.6% |
| Manual facemask ventilation | 5.5% |
| Leak fraction | 1.5% |
